# An online survey of Australian physicians reported practice with the off-label use of nebulised frusemide

**DOI:** 10.1186/1472-684X-11-6

**Published:** 2012-04-30

**Authors:** Phillip J Newton, Patricia M Davidson, Christine Sanderson

**Affiliations:** 1 Centre for Cardiovascular & Chronic Care, Faculty of Nursing, Midwifery & Health, University of Technology Sydney, PO Box 123 Broadway, Sydney, NSW, 2007, Australia; 2 Department of Palliative Care, Calvary Health Care, 91-111 Rocky Point Road, Beverley Park, Sydney, NSW, 2217, Australia

**Keywords:** Nebulised frusemide, Online survey, Off-label use

## Abstract

**Background:**

Off-label prescribing is common in palliative care. Despite inconsistent reports of the benefit of nebulised frusemide for breathlessness, its use continues to be reported.

**Methods:**

An online survey was emailed to 249 members of the Australian and New Zealand Society of Palliative Medicine to estimate the use of nebulised frusemide for breathlessness by Australian physicians involved in palliative care in the previous 12 months.

**Results:**

There were 52/249 (21%) respondents to the survey. The majority (44/52; 85%) had not prescribed nebulised frusemide in the previous 12 months. The most common (18/44; 43%) reason for not prescribing nebulised frusemide was a belief that there was not enough evidence to support its use. Whilst only a few respondents (8/52; 15%) reported having used nebulised frusemide, all that had used it thought there was at least some benefit in relieving breathlessness.

**Conclusion:**

This report adds to the series of case studies reporting some benefit from nebulised frusemide in relieving breathlessnes.

## Background

Off-label prescribing also known as unlabelled or unapproved prescribing occurs when an approved medication is used in a way that is not included or disclaimed in the product information brochure.[1] Off-label use may occur if the agent is prescribed in a dose, route, indication or age group for which the agent is not registered with the appropriate authority. The off-label prescribing of medicines may occur in as many as 20–40% of adults [2,3]. The extent to which off-label prescribing is based on good clinical data is also concerning. A recent study of over 150 million off-label prescriptions found that 73% had little if any scientific evidence to support the prescription [2]. Off-label prescribing is more common in some specialties or patient population than others [4]. In the relatively new specialty of palliative care, off-label prescribing occurs commonly because either the dose or the route of administration or pharmacological effects were not included in the original product label [4]. Monitoring these trends is important in documenting practice trends and the need for determining future empirical investigations.

Frusemide, a common loop diuretic is primarily used for the removal of excess fluid and has been approved to be given as an oral solution, tablet or intravenously. Primarily used for intractable breathlessness in advanced disease despite optimal treatment, nebulised frusemide has anecdotally been shown to be a useful therapeutic for breathlessness [5-7]. Both animal and human studies identified several possible mechanisms for the action of nebulised frusemide including enhanced pulmonary receptor activity, suppression of the pulmonary irritant activity, and vasodilatation although there is no acute haemodynamic effect [8,9] Whilst the early case-studies of nebulised frusemide suggested its use may be beneficial for breathlessness at the end-of-life, [5-7] two small subsequent randomized controlled trials failed to show benefit in people with breathlessness as a result of advanced cancer [10,11]. Despite these trials, the use of nebulised frusemide continues to be reported in the literature [12,13].

### Aim

The aim of this study was twofold. Firstly, to assess the reported practice of off-label use of nebulised frusemide for breathlessness by Australian physicians working in palliative care in the previous 12 months and secondly, to determine the feasibility and acceptability of using online surveys for assessing practice usage and gathering case reports.

### Methods

The survey instrument ( Additional file 1) was an investigator developed brief questionnaire that sought to assess the prescribing of nebulised frusemide by Australian palliative care physicians. An introduction letter explaining the purpose of the survey and the link to survey was emailed to 249 Australian members of the Australian and New Zealand Society of Palliative Medicine (ANZSPM). The survey was conducted using the SurveyMonkey platform (http://www.surveymonkey.com) and remained open for six weeks.

The survey ascertained the basic demographics related to medical specialty, highest palliative care qualification and length of time working in palliative care. Respondents were then asked if they had prescribed nebulised frusemide in the previous 12 months. For those who had prescribed nebulised frusemide, they were asked a series of questions relating to their opinion of efficacy and prescribing practice of nebulised frusemide. To establish in more detail the use and perceived efficacy of nebulised frusemide, those who had prescribed nebulised frusemide were given the option to describe (basic demographics, diagnosis, co-morbidities, treatments previously used for breathlessness, and any agents co-prescribed and the impression of the efficacy) for two cases where they had prescribed nebulised frusemide. If respondents had not prescribed nebulised frusemide in the previous 12 months, they were asked the main reason why they had not prescribed it. Ethics approval was received from Curtin University (SON&M 12-2010). Data are reported as number (percentage) unless otherwise specified.

## Results

Over a six week period, 52 responses were received. The majority of respondents (41/52; 79%) to the survey were palliative care consultants with 38/52 (73%) having dual Fellowships to the Royal Australasian College of Physicians and/or the Chapter of Palliative Medicine. The seniority of the clinicians who answered the survey was also reflected in the median length of time the respondents had been working in palliative care (14 years; interquartile range [IQR] 6 to 17). Responses were received from all six states in Australia with the majority of responses (41/51; 80%) coming from the three most populated states (New South Wales, Victoria and Queensland). One respondent did not answer this item.

### Physician’s reported practice related to nebulised frusemide

**Figure 1:**
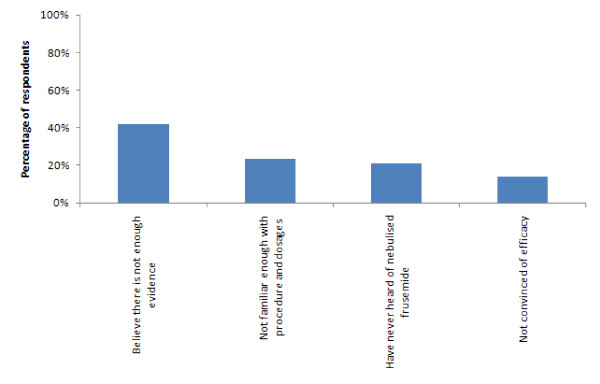
**Reasons for not using nebulised frusemide in the previous 12 months*(n = 43)***.

The majority of respondents (44/52; 85%) had not used nebulised frusemide in the previous 12 months. The main reason cited for not using it were that they (18/44; 41%) believed there was not enough evidence to support its use whilst only 6/44 (14%) were not convinced by its efficacy. Figure 1 provides the full results for this item. One respondent replied that they had not used nebulised frusemide in the previous 12 months because they were on maternity leave.

Only a small percentage of respondents (8/52; 15%) reported using nebulised frusemide in the previous 12 months. Based on their experience of nebulised frusemide, the majority (6/8; 75%) indicated that there was at least some use for nebulised frusemide. When asked where in the treatment hierarchy nebulised frusemide should be used, the majority (6/8; 75%) said that it should be used as a fourth line treatment.

When asked about the frequency of dosing that the respondents had prescribed nebulised frusemide, most had prescribed it as either three times daily (2/8; 25%) or four times daily (3/8; 38%). Two respondents (2/8; 25%) responded that they had only prescribed nebulised frusemide as a PRN medication. Two respondents (2/3; 67%) who had prescribed nebulised frusemide as a four times daily prescription had also prescribed nebulised frusemide as a PRN medication. A PRN prescription was also reported by a respondent (1/2; 50%) who had prescribed nebulised frusemide as a three times daily prescription. All respondents (8/8; 100%) reported that they had prescribed 20 mg of nebulised frusemide.

### Case series

The cases of prescribed nebulised frusemide in the previous 12 months by the respondents are presented in Table 1. Half of the cases reported were patients with a primary diagnosis of cancer (5/10; 50%) with the remaining having either chronic cardiac disease or chronic respiratory disease. Two cases (20%) reported a primary diagnosis as both chronic obstructive pulmonary disease and lung cancer. Pleural effusion and heart failure were the most common (3/10; 30%) problems causing breathlessness.

**Table 1:** Details of cases recalled by physicians where nebulised frusemide was used

	**Age**	**Sex**	**Primary diagnosis**	**Main problem causing breathlessness**	**Previous therapies for breathlessness**	**Impression of clinical efficacy**	**Comments**
Case 1	70	Female	NSCL cancer with COPD	Bronchial obstruction	Oxygen, Opioids, Steroids, Benzodiazepines, Bronchodilators, Antibiotics	Possible improvement	No comments provided
Case 2	78	Male	Pulmonary oedema	Cardiac failure	Oxygen, Opioids, Benzodiazepines	Unsure of any benefit	No comments provided
Case 3	60	Female	CRC, lung metastases, pleural effusion, lymphangitis	Pleural effusion, lymphangitis	Oxygen, Opioids, Steroids, Benzodiazepines, Bronchodilators, Drainage of pleural effusion	Possible improvement	No comments provided
Case 4	84	Male	Respiratory failure, History of AF & cardiomyopathy	Pulmonary fibrosis and some mild LVF	Oxygen, Opioids, Nebulised atrovent, Low dose midazolam	Obvious improvement	No comments provided
Case 5	70’s	Male	COPD, Lung cancer	COPD, Lung metastases	Oxygen, Opioids, Steroids, Benzodiazepines, Bronchodilators, Antibiotics	Possible improvement	No comments provided
Case 6	65	Male	Not reported	Not reported	Oxygen, Opioids, Steroids, Benzodiazepines	Possible improvement frusemide	No comments provided
Case 7	60’s	Female	Stage IV NSCLC	Cardiac failure	Oxygen, Opioids, Steroids, Benzodiazepines, Bronchodilators, Antibiotics	Possible improvement	No comments provided
Case 8	37	Female	Lung cancer	Local disease, airway compromise, effusion	Oxygen, Opioids, Steroids, Bronchodilators	Obvious improvement	She was quite anxious and benzo’s used helped significantly but further improvement seen after adding lasix as well
Case 9	72	Female	Metastatic vulval cancer	Pleural effusion	Oxygen, Opioids, Steroids, Glycopyrolate	Obvious improvement	Within 20 minutes, patient came out of unconsciousness. I am considering using now as first line with atrovent when acute episode occurs after chronic control.
Case 10	70’s	Male	Respiratory failure	End stage COPD	Oxygen, Opioids, Steroids, Benzodiazepines, Bronchodilators, Antibiotics	Obvious improvement	Addition of nebulised frusemide improved difficult dyspnea, where all other modalities had been tried;

*NSCL* Non-small cell carcinoma of the lung; *COPD* Chronic obstructive pulmonary disease; *CRC* colorectal cancer; *AF* atrial fibrillation; *IHD*: Ischemic heart disease.

A number of therapies were reported as having been used prior to nebulised frusemide with all (10/10; 100%) having previously used oxygen and opioids. The majority had also been previously prescribed steroids (8/10; 80%) and benzodiazepines (7/10; 70%). A number of therapies were co-prescribed with nebulised frusemide: oxygen (8/10; 80%), opioids (8/10; 80%), steroids (6/10; 60%), benzodiazepines (7/10; 70%) and bronchodilators (5/10; 50%). 

Most respondents reported that there was at least a possible improvement (5/10; 50%) with 4/10 (40%) of respondents reporting that there was an obvious improvement associated with the administration of nebulised frusemide. One respondent (10%) was unsure if there was any improvement associated with the use of nebulised frusemide.

### Survey acceptability

All respondents were asked to rate the level of difficulty completing the survey on a five point scale (not difficult at all – very difficult). The majority of respondents (47/51; 91%) selected that the survey was not difficult to complete whilst the remaining 4 (8%) respondents selected that it was a bit difficult to complete. One respondent (1/52; 2%) did not answer this item.

Respondents rated the usefulness of using an online survey to identify the prescribing patterns on a five point scale (not useful at all – very useful). All respondents reported that an online survey is at least somewhat useful with the majority saying this method is useful (33/51; 65%) or very useful (12/51; 24%). One (1/52; 2%) respondent did not complete this item.

When asked would they be willing to participate in a similar survey in the future, almost all respondents (47/50; 94%) said that they would be at least likely to participate again. Only one respondent (1/52; 2%) said they would be unlikely to participate in a similar survey again. Two (4%) respondents did not complete this item.

## Discussion

The majority of respondents did not use nebulised frusemide in the previous 12 months with the most common reason being that they (18/44, 41%) did not believe there is enough evidence to support its use. Whilst the early case series [5-7] reported indicated nebulised frusemide may be a useful novel therapy for breathlessness in advanced disease, two subsequent randomized trials have failed to show the benefit of nebulised frusemide over placebo in people with advanced cancer who remained breathless despite treatment with more established therapies [10,11]. Of those who had not used nebulised frusemide 7/44 (16%) had used it at some point previously but either had not been convinced of its efficacy (6/44; 14%) or the clinical scenario had not arisen where they would prescribe it (1/44; 2%). It is not known how long ago these respondents had prescribed nebulised frusemide.

All respondents who reported prescribing nebulised frusemide in the previous 12 months (8/52, 15%) reported that they had used 20 mg of frusemide. One respondent stated that following a loading dose and “if the patient is very wet and able to cope” they would use 40 mg. This is in keeping with the reported case series [5-7] and clinical trials [10,11]. The failure of the clinical trials to show the benefit of benefit of nebulised frusemide over placebo has seen some to conclude that the benefit reported in the initial case series was the result of a placebo effect [10].

Although there is limited evidence at best for the use of nebulised frusemide for breathlessness due to advanced disease, it is still being used by physicians even if only in limited circumstances [12,13]. Like much of the breathlessness literature, both clinical trials and many of the case-series were conducted in people with malignant disease. However, this survey has shown that some physicians are also using nebulised frusemide in non-malignant conditions. It was clear based on the responses that its use was reserved once a number of other therapies, particularly opioids and oxygen had been tried but had failed to provide sufficient symptomatic relief. These data also underscore the common and sometimes refractory burden of breathlessness [14].

### Limitations

Our response rate 52/249 (21%) is comparable to other reported online physician surveys, [15] although some have reported much higher [16] and lower [17] response rates. Whilst traditional mail, fax or telephone surveys report higher response rates than online surveys, [15,16,18] we felt that the cost and time associated with these modes of delivery prohibited their use in this survey. It is likely that the response rate would have been improved had we been able to send a reminder to the respondents about the survey [19]. The use of ANZSPM to distribute the invitation and the link to the survey to its members limited the risk that the survey was answered by a respondent who was either not a physician (as ANZPM membership is only available to physicians) and involved in the palliative care of patients. Some authors have also suggested that younger physicians are more likely to complete online surveys than older physicians [15] however the majority (41/52; 79%) of respondents in this survey were senior physicians with a median of 14 (IQR 6 to 17) years working in palliative care. It may be that the generation of physicians that were more likely to respond to the web-based surveys in these early reports are now the senior clinicians willing to participate in online surveys. Because of the mode of delivery of the survey, respondents were limited to ANZSPM members with an email address.

The cases reported in Table 1 relied on the clinician’s recall of the details and so there is the potential for recall bias. This potential was one of the reasons why we only asked respondents to report the use of nebulised frusemide in the previous 12 months. Respondents who completed the case details were asked if they would be willing to validate their responses by reviewing the notes of the reported cases and re-enter the information which would have allowed us to assess recall bias. Unfortunately, only 3/8 (38%) respondents who reported the use of nebulised frusemide said they would be willing to do this. One respondent (33%) who was willing to review the notes said they had subsequently moved interstate and no longer had access to the case notes. The remaining two respondents did not log back into the survey to enter the details. We were unable to send reminders to these clinicians to re-enter the information after reviewing the case notes. Future studies should try to capture this information.

### Future research

As palliative care matures as a medical specialty, online surveys provide a relative quick and easy opportunity to better understand the prescribing practices of its professionals. The majority of respondents in this survey found it easy to use and indicated they would be willing to participate in similar surveys in future. Online surveys can and have been used to quickly gauge the opinions of a range of physicians on a topic [20] which has then directly contributed to the design and implementation of clinical research [21]. Given the limited, although ever expanding evidence for treatments used in palliative care, online surveys are a valuable method to determine the research priorities for the specialty. Undeniably the state of equipoise for many in the use of nebulised frusemide demands well designed clinical trials in target populations using reliable and valid measures of outcome.

## Conclusion

This online survey shows that despite only anecdotal evidence of benefit and two small, negative randomized controlled trials, nebulised frusemide continues to be used by some Australian physicians working in palliative care. The continued use of nebulised frusemide in people with intractable breathlessness despite optimal treatment likely reflects the limited therapeutic options available. Research must continue to determine if nebulised frusemide is effective and in which clinical situations.

## Competing interests

The authors declare that they have no competing interests.

## Authors’ contributions

PJN: Study design, data analysis and interpretation, manuscript preparation. PMD: Study design, interpretation of data, manuscript preparation. CS: Study design, interpretation of data, manuscript preparation. All authors read and approved the final manuscript.

## Pre-publication history

The pre-publication history for this paper can be accessed here:

http://www.biomedcentral.com/1472-684X/11/6/prepub

## Supplementary Material

Additional file 1 The use of nebulised frusemide in Australian palliative care survey. Click here for file
