# Supplementary material for: An online survey of Australian physicians reported practice with the off-label use of nebulised frusemide
Source: BMC Palliat Care. 2012 Apr 30;11:6. doi: 10.1186/1472-684X-11-6 (PMC3422185; doi:10.1186/1472-684X-11-6)
Supplement: Additional file 1 — The use of nebulised frusemide in Australian palliative care survey. [file 1472-684X-11-6-S1.pdf]

The use of nebulised frusemide in Australian palliative care

1. Background to this survey

Dear colleague

Thank you for taking the time to complete this survey. This study will have been explained to you in the email providing the link to this survey.

The use of nebulised frusemide has been proposed for dyspnoea, and is currently prescribed by some palliative care clinicians. Currently, there is not robust evidence to support or refute this practice.

This brief online survey will collect information about the current use of nebulised frusemide by Australian palliative care clinicians.

We would very much appreciate your time in completing the survey, which should take about 10 minutes. If you are interested in receiving further information about nebulised frusemide, you can provide your email address at the end of the survey and additional survey will be sent to you.

We are also interested in obtaining your views of this method of using brief online surveys as a way to collect information about prescribing practices in palliative care.

This survey is being undertaken by ImPACCT (Improving Palliative Care through Clinical Trials) which is the New South Wales (NSW) collaborative clinical trials group in palliative care

Information from this survey will assist us in planning future clinical trials.

Dr Christine Sanderson
Dr Phillip Newton
Professor Patricia Davidson
for ImPaCCT (Improving Palliative Care through Clinical Trials)

The use of nebulised frusemide in Australian palliative care

1. Participant Consent Statement

Ethics approval No: SON&M12-2010

Chief Investigators: Dr Christine Sanderson, Dr Phillip Newton, Professor Patricia Davidson for NSW Palliative Care Clinical Trials Collaborative Research Group (ImPACCT)

I am making a decision to voluntarily participate in the research project named above. By ticking the box below I indicate that I have read and understood the 'Participant Information Sheet' provided.

I understand that:

1. Participation involves completing an on-line questionnaire which will take approximately 5-10 minutes to complete
2. I can withdraw from the study at any time by contacting Dr Christine Sanderson on 02 95533111
(telephone) or christinersanderson@gmail.com

My withdrawal will not in any way affect my relationship with the study investigators.
3. If you have any concerns about the way in which this research is being conducted, you may contact the Manager, Research Ethics at Curtin University of Technology on (08)92662784 quoting Curtin University Ethics Approval Number: SON&M12-2010
4. The research study is strictly confidential and no information about me will be used in any way that reveals my identity

◯ I hereby agree to participate in this research study

◯ I do not wish to participate in this study

2. Some information about you

This section is designed to obtain some information regarding your professional status

The use of nebulised frusemide in Australian palliative care

*1. Your current role is

- General practitioner
- Resident medical officer working in palliative care
- Career medical officer working in palliative care
- Palliative care consultant
- Paliative care registrar
- Other (please specify)

2. What is your highest qualification in palliative care?

- No formal qualification
- Clinical diploma
- Advanced trainee in palliative care
- Master of palliative care
- Fellow of the Chapter of Palliative Medicine and/or FRACP
- Dual fellowship: Fellowship of Royal Australasian College of Physicians with Advanced training in both Palliative Medicine and Medical Oncology

*3. How many years of palliative care experience do you have?

4. Where do you work?

- New Zealand
- Queensland
- New South Wales
- ACT
- Victoria
- Tasmania
- South Australia
- Northern Territory
- Western Australia

3. Nebulised frusemide use

*1. Have you prescribed nebulised frusemide for dyspnoea in the last 12 months?

- Yes
- No

4. If you have not prescribed nebulised frusemide

We are interested in exploring your reasons for not using nebulised frusemide

1. If you have not used nebulised frusemide in the last 12 months, please tick all of the following reasons why you have not done so

- ◯ Have never heard of administering frusemide via the nebulised route
- ◯ Aware of the potential of administration but not familiar enough with procedure and dosages
- ◯ Believe there is not enough evidence for nebulised frusemide
- ◯ Have used it previously and not been convinced of its effectiveness
- ◯ Have used it and noted adverse effects or other problems (please describe)

2. Please provide any other information that may explain why you have not used nebulised frusemide in the last 12 months.

5. Nebulised frusemide prescribing

1. If you have used nebulised frusemide- what is your perception of its use in relieving dyspnoea

- ◯ no use
- ◯ some use
- ◯ unsure
- ◯ useful
- ◯ very useful

2. What do you think should be the place of nebulised frusemide in treatment?

- ◯ First line
- ◯ Second line
- ◯ Third line
- ◯ Fourth line

***3. What frequency of administration do you prescribe nebulised frusemide? (select all that apply)**

- [ ] Daily
- [ ] Twice daily
- [ ] Three times daily
- [ ] Four times daily
- [ ] PRN
- [ ] Other (please specify)

***4. What dose of nebulised frusemide do you normally prescribe? (select all that apply)**

- [ ] 20 mg
- [ ] 30 mg
- [ ] 40 mg
- [ ] 60 mg
- [ ] 80 mg
- [ ] Other (please specify)

6. Case A

Please try to recall the last two patients you prescribed nebulised frusemide for and describe them below.

1. Age

2. Gender

- ( ) Male
- ( ) Female

3. Diagnosis

4. Main problem causing dyspnoea (please be as precise as possible, eg pulmonary fibrosis, or cardiac failure, or pleural effusion, etc etc)

5. Main comorbidities

6. Other treatments already tried for dyspnoea:

- [ ] Oxygen
- [ ] Opioids
- [ ] Steroids
- [ ] Benzodiazepines
- [ ] Bronchodilators
- [ ] Antibiotics
- [ ] Non-invasive ventilation
- [ ] None
- [ ] Can not recall
- [ ] Other (please specify)

7. Please select any other treatments co-prescribed with nebulised frusemide:

- [ ] Oxygen
- [ ] Opioids
- [ ] Steroids
- [ ] Benzodiazepines
- [ ] Bronchodilators
- [ ] Antibiotics
- [ ] Non-invasive ventilation
- [ ] None
- [ ] Can not recall
- [ ] Other (please specify)

8. Clinical impression of effectiveness in this case:

- [ ] Obvious improvement associated with use of nebulised frusemide
- [ ] Possible improvement associated with use of nebulised frusemide
- [ ] No improvement

Any other comments (for instance - time to onset of effect, duration of the effect, any adverse effects, or problems with administration)?

7. Case B

1. Age

2. Gender

- ( ) Male
- ( ) Female

3. Diagnosis

4. Main problem causing dyspnoea (please be as precise as possible, eg pulmonary fibrosis, or cardiac failure, or pleural effusion, etc etc)

5. Main comorbidities

6. Other treatments already tried for dyspnoea:

- [ ] Oxygen
- [ ] Opioids
- [ ] Steroids
- [ ] Benzodiazepines
- [ ] Bronchodilators
- [ ] Antibiotics
- [ ] Non-invasive ventilation
- [ ] None
- [ ] Can not recall
- [ ] Other (please specify)

7. Please select any other treatments co-prescribed with nebulised frusemide:

- [ ] Oxygen
- [ ] Opioids
- [ ] Steroids
- [ ] Benzodiazepines
- [ ] Bronchodilators
- [ ] Antibiotics
- [ ] Non-invasive ventilation
- [ ] None
- [ ] Can not recall
- [ ] Other (please specify)

8. Clinical impression of effectiveness in this case:

- [ ] Obvious improvement associated with use of nebulised frusemide
- [ ] Possible improvement associated with use of nebulised frusemide
- [ ] No improvement

Any other comments (for instance - time to onset of effect, duration of the effect, any adverse effects, or problems with administration)?

8. Some brief comments on this survey

We are interested in obtaining your views on this survey.

The use of nebulised frusemide in Australian palliative care

1. How important do you think it is to obtain information about drug prescribing in palliative care practice?

- ◯ not important
- ◯ somewhat important
- ◯ important
- ◯ very important

2. How difficult was this survey to complete?

- ◯ not difficult at all
- ◯ a bit difficult
- ◯ difficult
- ◯ very difficult

3. Do you think the method of an on-line survey is useful to identifying prescribing patterns?

- ◯ not useful at all
- ◯ somewhat useful
- ◯ useful
- ◯ very useful

4. If you completed the case studies as part of this survey please identify how difficult it was for you to recall the information?

- ◯ did not complete case study
- ◯ not difficult
- ◯ somewhat difficult
- ◯ difficult
- ◯ very difficult

Please provide comments if necessary

5. If you were asked to participate in a survey of this type in the future, how likely would you be to participate?

- ◯ not likely at all
- ◯ somehat likely
- ◯ likely
- ◯ very likely

***6. Are you willing in help us validate the accuracy of the case studies described?**

- ◯ I did not describe any cases
- ◯ No
- ◯ Yes

9. Case study validation

We are interested in assessing the accuracy of the case studies described by asking you to review the clinical notes of the patients described and complete the same questions.

If you are interested in completing this section, simply press the "Exit this survey" button in the top right hand corner of the page.

After reviewing the clinical notes, click on the link in the email that you received to start this survey and you will be brought back to this page and will be able to answer the remaining questions.

10. Case A - notes reviewed

Please complete the following questions based after you have reviewed the clinical notes

1. Age

2. Gender

- ◯ Male
- ◯ Female

3. Diagnosis

4. Main problem causing dyspnoea (please be as precise as possible, eg pulmonary fibrosis, or cardiac failure, or pleural effusion, etc etc)

5. Main comorbidities

6. Other treatments already tried for dyspnoea:

- ☐ Oxygen
- ☐ Opioids
- ☐ Steroids
- ☐ Benzodiazepines
- ☐ Bronchodilators
- ☐ Antibiotics
- ☐ Non-invasive ventilation
- ☐ None
- ☐ Can not recall
- ☐ Other (please specify)

7. Please select any other treatments co-prescribed with nebulised frusemide:

- ☐ Oxygen
- ☐ Opioids
- ☐ Steroids
- ☐ Benzodiazepines
- ☐ Bronchodilators
- ☐ Antibiotics
- ☐ Non-invasive ventilation
- ☐ None
- ☐ Can not recall
- ☐ Other (please specify)

8. Clinical impression of effectiveness in this case:

- ☐ Obvious improvement associated with use of nebulised frusemide
- ☐ Possible improvement associated with use of nebulised frusemide
- ☐ No improvement

Any other comments (for instance - time to onset of effect, duration of the effect, any adverse effects, or problems with administration)?

9. Was there any evidence from the notes to support answer regarding clinical effectiveness (eg reduction in the number of breakthroughs, documented comments by patient or other team members, SAS results for the symptom)?

- ○ No
- ○ Yes (please specify)

10. Was there any documentation of adverse events related to nebulised frusemide use?

- ○ No
- ○ Yes (please specify)

11. Case B - notes reviewed

Please complete the following questions based after you have reviewed the clinical notes

1. Age

2. Gender

- ◯ Male
- ◯ Female

3. Diagnosis

4. Main problem causing dyspnoea (please be as precise as possible, eg pulmonary fibrosis, or cardiac failure, or pleural effusion, etc etc)

5. Main comorbidities

6. Other treatments already tried for dyspnoea:

- ☐ Oxygen
- ☐ Opioids
- ☐ Steroids
- ☐ Benzodiazepines
- ☐ Bronchodilators
- ☐ Antibiotics
- ☐ Non-invasive ventilation
- ☐ None
- ☐ Can not recall
- ☐ Other (please specify)

7. Please select any other treatments co-prescribed with nebulised frusemide:

- ☐ Oxygen
- ☐ Opioids
- ☐ Steroids
- ☐ Benzodiazepines
- ☐ Bronchodilators
- ☐ Antibiotics
- ☐ Non-invasive ventilation
- ☐ None
- ☐ Can not recall
- ☐ Other (please specify)

8. Clinical impression of effectiveness in this case:

- ☐ Obvious improvement associated with use of nebulised frusemide
- ☐ Possible improvement associated with use of nebulised frusemide
- ☐ No improvement

Any other comments (for instance - time to onset of effect, duration of the effect, any adverse effects, or problems with administration)?

9. Was there any evidence from the notes to support answer regarding clinical effectiveness (eg reduction in the number of breakthroughs, documented comments by patient or other team members, SAS results for the symptom)?

- ◯ No
- ◯ Yes (please specify)

10. Was there any documentation of adverse events related to nebulised frusemide use?

- ◯ No
- ◯ Yes (please specify)

12. Are you interested in participating in an email group?

Subscribing to this email list will allow access to further information regarding nebulised frusemide and in the future potential opportunities to participate in clinical studies.

1. Please provide your email address or if you want to provide your contact details seperate to this questionnaire please email phillip.newton@uts.edu.au
